# Polyhydroxy‐Decorated 2D Covalent Organic Framework with Imine Active Sites toward Efficient Electrocatalytic Oxygen Reduction Reaction: Experimental and Theoretical Insights

**DOI:** 10.1002/asia.202500592

**Published:** 2025-07-08

**Authors:** Manisha Das, Thakur Rochak Kumar Rana, Santanu Chand, Sumit Kumar, Takaya Ogawa, Laurent Billon, Tokuhisa Kawawaki, Sabuj Kanti Das, Yuichi Negishi

**Affiliations:** ^1^ Graduate School of Energy Science Kyoto University Yoshida‐honmachi Sakyo‐ku Kyoto 606–8501 Japan; ^2^ Department of Chemistry Indian Institute of Technology Bombay, Powai Mumbai Maharashtra 400076 India; ^3^ Department of Applied Chemistry Graduate School of Engineering The University of Tokyo Bunkyo‐ku Tokyo 113–8656 Japan; ^4^ Department of Physics N.R.E.C. College Khurja Uttar Pradesh 203131 India; ^5^ Bio‐Inspired Materials Group: Functionalities & Self‐Assembly Universite de Pau et des Pays de l'Adour E2S UPPA, UPPA/CNRS, IPREM UMR 5254, 2, Avenue du Président Angot Pau 64053 France; ^6^ Institute of Multidisciplinary Research for Advanced Materials Tohoku University Aoba‐ku Sendai 980–8577 Japan; ^7^ Research Institute for Science & Technology Tokyo University of Science Tokyo 162–8601 Japan

**Keywords:** Covalent organic frameworks (COFs), Heteroatom rich, Metal‐free electrocatalyst, Oxygen reduction reaction (ORR), Theoretical mechanistic analysis

## Abstract

The development of metal‐free electrocatalysts for the oxygen reduction reaction (ORR) is crucial for next‐generation energy technologies, offering cost reduction, improved stability, and independence from scarce noble metals. Covalent organic frameworks (COFs) have emerged as promising candidates due to their tunable porosity, high surface area, and structural versatility. In this study, we report a hydroxyl‐functionalized enamine‐ and imine‐linked two‐dimensional (2D) COFs (TFPh_DHPh_COF) as an efficient ORR electrocatalyst in alkaline media. The enamine (─C─NH─) linkages enhance structural integrity through hydrogen‐bonding interaction, whereas imine (─C═N─) linkages contribute to electron density modulation, stabilizing key intermediates and facilitating a favorable four‐electron ORR pathway. Natural bond orbital (NBO) analysis from computational study, reveals a partial positive charge (*C* ^δ⁺^ = 0.12) at the active site, promoting O_2_ activation via end on binding and weakening the O═O bond (Pauling model). Electrochemical evaluation demonstrates a 0.72 V vs reversible hydrogen electrode (RHE) half‐wave potential and exceptional durability, outperforming many metal‐free catalysts under basic media. This study underscores the potential of functionalized COFs as a scalable and sustainable alternative to metal‐based catalysts for fuel cells and metal–air batteries.

## Graphical abstract

Hydroxyl‐functionalized enamine/imine‐linked two‐dimensional (2D) COFs (TFPh_DHPh_COF) exhibits efficient and durable metal‐free ORR activity in alkaline media via a favorable 4e⁻ pathway. Enhanced charge transfer and O_2_ activation arise from tailored linkages and active‐site charge distribution.

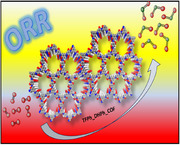

## 1 Introduction

The growing global energy demand and environmental concerns associated with fossil fuel consumption have intensified the search for sustainable and efficient energy conversion technologies.^[^ ^1^, ^2^ ^]^ Fossil fuels, which currently dominate the global energy landscape, contribute significantly to greenhouse gas emissions, leading to climate change, air pollution, and resource depletion.^[^ ^3^ ^]^ Additionally, their finite nature and fluctuating availability necessitate the transition toward cleaner alternatives. Among various renewable energy solutions, electrochemical energy conversion technologies, such as fuel cells and metal–air batteries, have gained significant attention due to their high energy efficiency, low environmental impact, and ability to directly convert chemical energy into electricity.^[^ ^4^, ^5^ ^]^ However, their widespread commercialization is hindered by the sluggish kinetics of the oxygen reduction reaction (ORR) at the cathode, which significantly limits overall system efficiency, given that ORR is central to technologies in industrial electrochemical processes, enhancing its kinetics holds significant implications for both portable and grid‐scale energy applications.^[^ ^6^, ^7^, ^8^ ^]^ To overcome this challenge, electrocatalysts are required to accelerate the ORR and reduce energy losses. Traditionally, platinum (Pt)‐based materials have been employed as benchmark catalysts due to their superior activity and selectivity for ORR.^[^ ^9^ ^]^ However, their high cost, scarcity, and susceptibility to poisoning by intermediates (such as CO and hydroxyl species) make them less sustainable for large‐scale deployment. Moreover, Pt‐based systems often suffer from poor durability in alkaline environments and limited tolerance to methanol crossover, further motivating the development of robust, nonmetal alternatives. Therefore, the development of cost‐effective, metal‐free, and high‐performance electrocatalysts has become a critical research focus in the pursuit of clean energy technologies. Among the emerging alternatives, covalent organic frameworks (COFs) have garnered significant attention due to their high surface area, tunable porosity, and chemically versatile framework.^[^ ^10^, ^11^ ^]^ Unlike conventional porous materials, COFs offer atomic precision in building block arrangement, which enables systematic tuning of their electronic and catalytic behavior through bottom–up design. First reported by Yaghi and coworkers in 2005, COFs are crystalline porous polymers with precisely defined periodic structures that facilitate efficient charge transport and active site exposure.^[^ ^12^ ^]^ Enhancing the active site functionality in COFs to improve their performance in ORRs is still an area of ongoing investigation with significant untapped potential. Active sites are specific regions within a catalyst where reactant molecules, such as oxygen in the ORR process, adsorb and undergo chemical transformation. In metal‐free COFs, these active sites often arise from heteroatoms (like nitrogen, oxygen, or sulfur) and conjugated linkages (such as imine groups) that modulate local electronic environments. Imine‐linked COFs, formed through Schiff‐base condensation reactions between aldehydes and amines, are among the most extensively studied due to their synthetic simplicity, structural stability, and extended π‐conjugation. The imine (─C═N─) linkages offer electron‐rich sites that facilitate O_2_ adsorption, weaken the O═O bond, and promote electron transfer, all of which are crucial for enhancing ORR kinetics. These frameworks also allow modular design, enabling the incorporation of donor–acceptor motifs, redox‐active units, or hydrogen‐bonding groups that fine‐tune the local electronic environment and improve catalytic performance. Beyond electrocatalysis, imine‐linked COFs have demonstrated versatility in gas storage, molecular separations, and photoredox catalysis, underscoring their relevance across a wide spectrum of clean energy and environmental technologies.^[^ ^13^, ^14^ ^]^ Tailoring the type, distribution, and accessibility of their active sites is key to optimizing electrocatalytic performance. Recently, various COF‐based materials have been explored for the ORR, including heteroatom‐doped COFs, where the incorporation of nitrogen, boron, phosphorus, or sulfur enhances electronic density and promotes O_2_ adsorption, improving catalytic performance through the formation of graphitic‐N and pyridinic‐N active sites.^[^ ^15^, ^16^, ^17^ ^]^ Similarly, metal‐doped COFs, such as Fe, Co, or Mn‐incorporated frameworks, exhibit M‐N‐C active sites that mimic Pt‐based catalysts, demonstrating high ORR selectivity and stability.^[^ ^18^, ^19^ ^]^ Additionally, π‐conjugated COFs improve better charge transportation via extended delocalization of π‐electron cloud through the whole structure, which enhanced catalytic activity observed in COFs hybridized with conductive carbon materials.^[^ ^20^, ^21^ ^]^ Despite their structural advantages, the electron transport efficiency within these frameworks through the porous channel (like two‐dimensional (2D) channel) and the availability of well‐defined active sites remain unconstrained, thereby facilitate their electrocatalytic capabilities. Tailoring pore channel orientation and confinement environments has proven effective in steering mass transport and reactant accessibility, significantly impacting ORR kinetics.^[^ ^22^ ^]^ Moreover, the choice of linkage chemistry plays a pivotal role in tuning both electronic structure and chemical stability. Linkages such as imine, hydrazone, and β‐ketoenamine offer different degrees of conjugation and rigidity, directly influencing charge transport and active site availability.^[^ ^23^ ^]^ The dimensionality of the COF architecture, 2D vs 3D (three‐dimensional) further affects the stacking mode, diffusion pathways, and electronic delocalization. 2D layered COFs often promote planar charge transport, whereas 3D frameworks offer improved surface exposure and isotropic diffusion.^[^ ^24^ ^]^ The ORR activity of COFs is governed by the nature and distribution of heteroatoms, electronic properties, and structural configuration. Imine‐linked donor–acceptor COFs offer tailored active sites, enhancing electron transport and catalytic efficiency. Accounting these factors, Dey et al. proposed choice of linkage chemistry and functional groups in COFs/COP (covalent organic polymers) which further refines their catalytic performance, with imine (─C═N─) linkages as the active sites, promoting charge delocalization and facilitating O_2_ adsorption and stabilizing reaction intermediates.^[^ ^10^, ^25^ ^]^ In case of ORR process, interaction of O_2_ on catalytic surface for O═O bond elongation which facilitates the bond dissociation step followed mainly two models, such a s Pauling model (end on interaction) and Yeager model (side‐on interaction).^[^ ^26^ ^]^ In another work, Dey et al. reported a triazine‐based COP for electrocatalytic ORR where the both models were followed toward O═O bond elongation and dissociation to achieve high half‐wave potential of 0.73V (vs RHE).^[^ ^26^ ^]^ These insights emphasize the importance of precisely engineered electronic environments within COFs to effectively mediate the O_2_ activation step, a critical determinant of ORR performance.

In this study, we report the rational design and synthesis of a new hydroxyl‐functionalized 2D COFs (TFPh_DHPh_COF) featuring both imine and aminal linkages. The COF was synthesized via a solvothermal polycondensation reaction between 2,4,6‐triformylphenol (TFPh) and 2,5‐diamino‐1,4‐benzenediol (DHPhDA). The resulting crystalline framework exhibits a well‐defined mesoporous architecture decorated with redox‐active heteroatoms, which facilitates enhanced electronic conductivity and promotes efficient mass and charge transport through its extended π‐conjugated backbone. The integration of hydroxyl groups and dual‐linkage chemistry not only introduces additional hydrogen‐bonding interactions to stabilize the framework but also enhances the density and accessibility of active sites. These active sites are strategically distributed within the 2D channels, enabling favorable end on O_2_ adsorption in accordance with the Pauling model, which is essential for promoting the direct four‐electron pathway in the ORR.^[^ ^27^ ^]^ This mechanism significantly reduces the formation of undesirable peroxide intermediates and improves reaction selectivity. Comprehensive electrochemical evaluations, supported by density functional theory (DFT) calculations, reveal that TFPh_DHPh_COF exhibits a positive onset potential, a high half‐wave potential (E_1/2_), and outstanding long‐term operational stability in alkaline media. These metrics are indicative of its superior catalytic performance, positioning it as a promising metal‐free alternative to conventional Pt‐based catalysts.

## 2 Results and Discussion

The solvothermal polycondensation reaction between a tripodal monohydroxy containing aldehyde (TFR) and a dihydroxy group functionalized diamine (DHPhDA) results a 2D COF which was successfully utilized toward metal free ORR. To understand the superiority of ─OH functionalized COF toward ORR, a controlled electrochemical study along with theoretical investigation were also performed using another ‐Me (methyl) functionalized COF (TFPhDMePh) structure. Synthesis and physical characterizations of control sample are provided in Supporting Information.

### 2.1 Synthesis of TFPh_DHPh_COF

**Figure 1:**
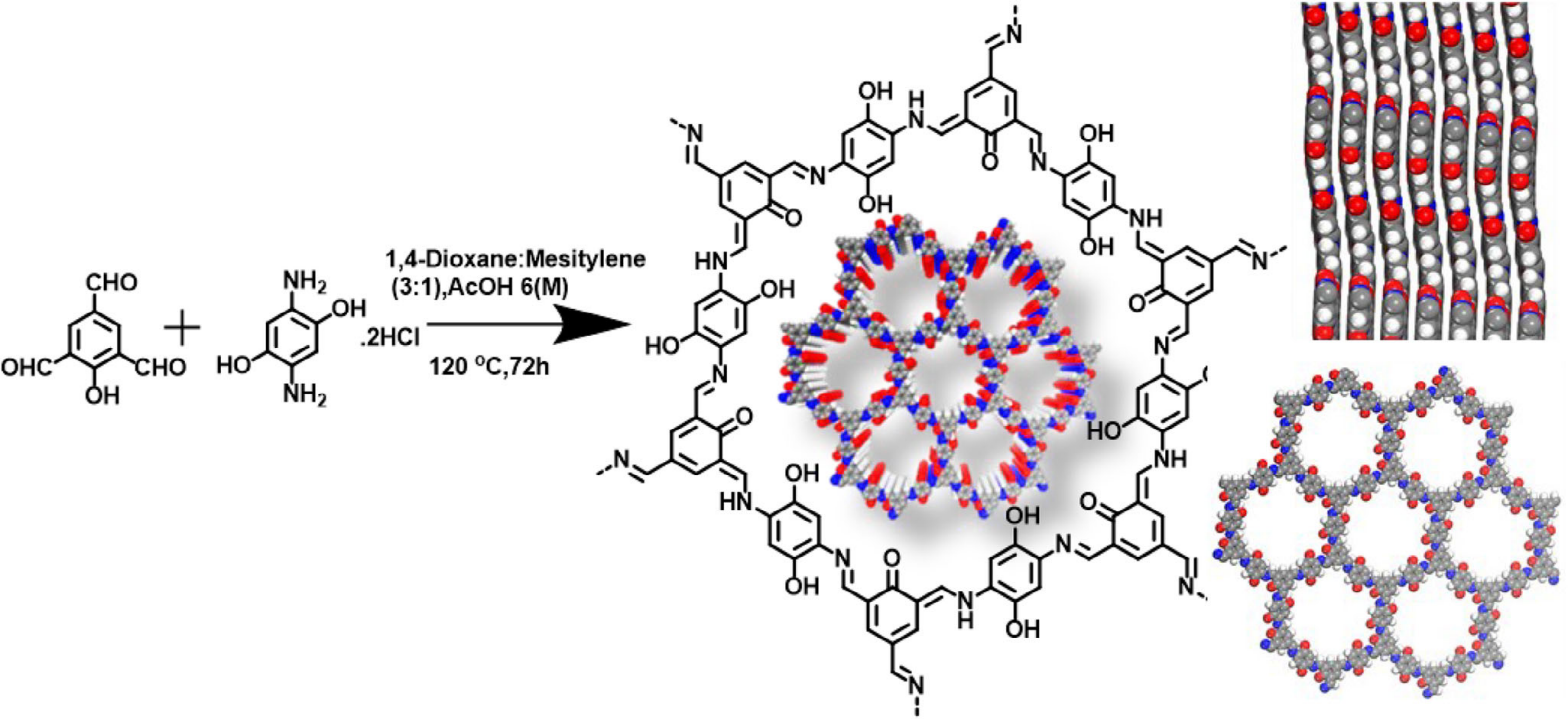
Schematic representation of TFPh_DHPh_COF synthesis, π–π stacking, and 2D extended view.

In a typical solvothermal COF synthesis procedure,^[^ ^28^ ^]^ 2,4,6‐triformylphenol (TFPh) (synthesis procedure reaction is in Figure S1) and 2,5‐diamino‐1,4‐dihydroxybenzene dihydrochloride (DHPhDA) were combined in a 2:3 molar ratio within a Schlenk tube. A mixture of 3 mL of 1,4‐dioxane and mesitylene (3:1) was then added, followed by the gradual addition of 0.2 mL of AcOH (6 M). To eliminate dissolved gases, the reaction mixture underwent three cycles of the Freeze–Pump–Thaw process. The sealed system was then heated at 120°C in an oil bath under static conditions. After three days, the reaction was allowed to cool to room temperature, and the resulting product was separated via filtration. The obtained solid was thoroughly washed with ethanol (EtOH), *N*,*N*‐dimethylformamide (DMF), tetrahydrofuran (THF), and acetone before being dried under vacuum at 110°C, yielding COF with an isolated yield of 74%. The resulting material exhibited a COF structure (Figure 1), designated as TFPh_DHPh_COF. This framework possesses β‐keto enamine (─C═C─NH─), phenolic (─OH), and imine (─C═N─) functional groups, capable of undergoing tautomerization between imine‐bonding linkage and ─OH group present in TFPh, as illustrated in Figure S2. ‐Me (methyl) functionalized COF was synthesized (see Supporting Information for details) following the same procedure where only DHPhDA was replaced by 2,5‐dimethyl‐1,4‐phenylenediamine (DMePhDA) as shown in Figure S3.

### 2.2 Electrochemical Measurements

All electrochemical measurements were conducted using a three‐electrode system, where an Ag/AgCl (3 M KCl) electrode served as the reference, a graphite rod (10 mm diameter) acted as the counter electrode, and a glassy carbon electrode (GCE, 3 mm diameter), rotating disk electrode (RDE, 5 mm diameter), or rotating ring‐disk electrode (RRDE, 5 mm diameter) functioned as the working electrode. Prior to experimentation, the electrolyte (0.1 M KOH) was saturated with oxygen by continuous bubbling of O_2_ gas for 30 min. The potential values recorded against Ag/AgCl (3 M KCl) were converted to the reversible hydrogen electrode (RHE) scale using the Nernst equation as depicted in Equation (1):

(1) $$E_{\mathrm{RHE}}\left(V\right)=E_{\mathrm{Ag}/\mathrm{AgCl}\;(3\;M\;\mathrm{KCl})}V+\left(0.058\times \mathrm{pH}\right)V+0.210\;V$$

### 2.3 Physical Characterizations

**Figure 2:**
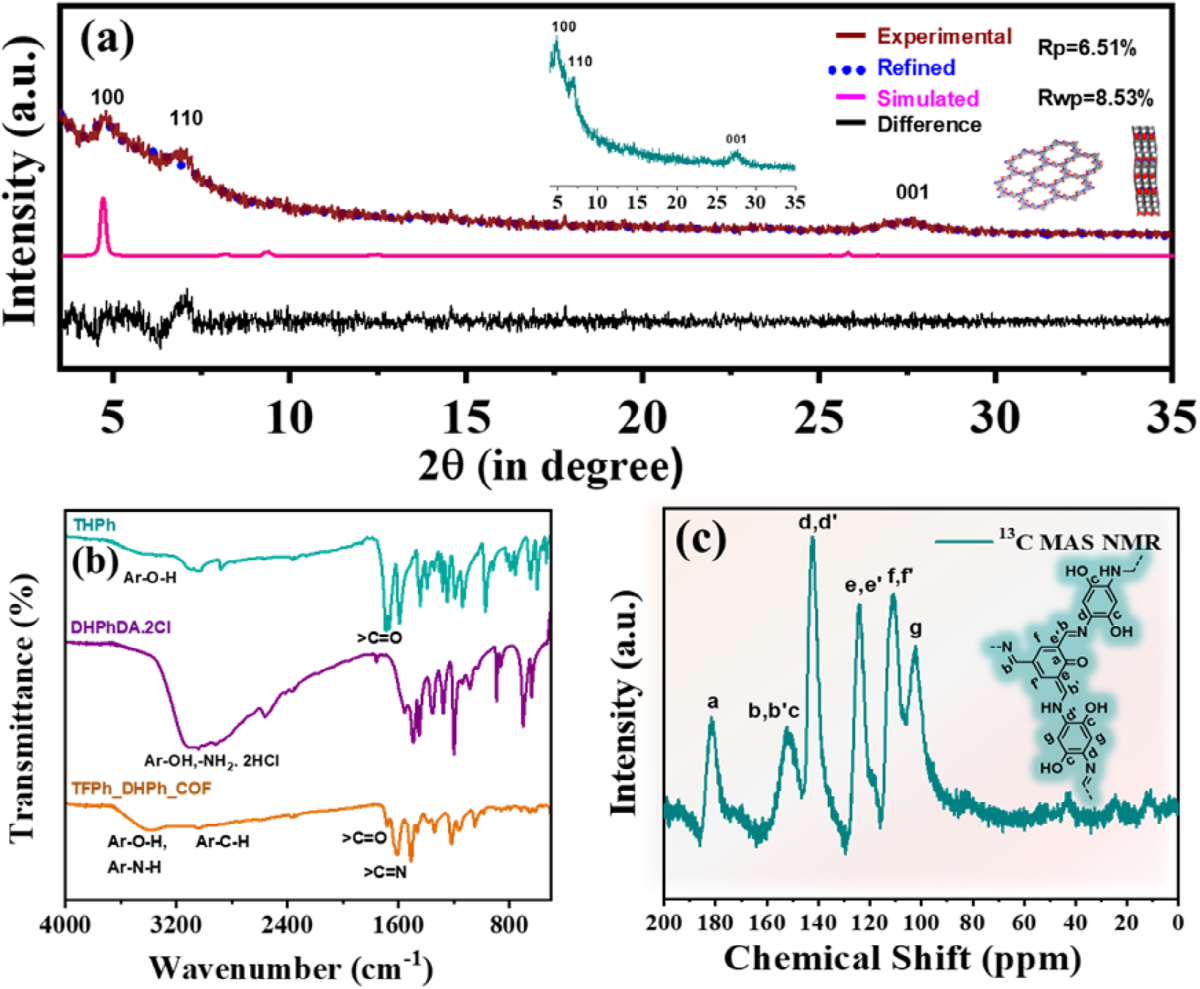
a) XRD analysis of as‐synthesized TFPh_DHPh_COF (inset) and corresponding Pawley refinement study. b) FTIR analysis of TFPh_DHPh_COF, THPh, and DHPhDA c) corresponding ^13^C MAS NMR of TFPh_DHPh_COF.

To identify the structural characteristics of the as‐synthesized TFPh_DHPh_COF, X‐ray diffraction (PXRD) analysis was conducted as depicted in Figure 2a. The COF possesses triclinic P1 space group along with a,b,c and α,β,γ values are found to be 22.7395, 22.8306, 3.6318 Å and 72.95°, 103.90°, and 123.39°, respectively. Unit cell parameters and fractional atomic coordinates of TFPh_DHPh_COF are summarized in Table S1. The experimental PXRD pattern exhibits distinct diffraction peaks corresponding to specific (hkl) values, confirming the crystalline nature of the material. The observed peaks at 2*θ* values of 4.7°, 6.6°, and 26.7° are attributed to the (100), (110), and (001) planes, respectively, indicating the ordered crystalline organic framework of TFPh_DHPh_COF. The broad diffraction peak in the range of 25°–30° is observed, which is characteristic of the π–π stacking interactions between the 2D layers of the COF, confirming the layered structure. The Pawley refinement analysis, as shown in the inset, aligns well with the simulated pattern, validating the structural model with refinement parameters of *R*_p_ = 6.51%. and *R*_wp_ = 8.53% and These results affirm the successful formation of crystalline TFPh_DHPh_COF with well‐defined stacking and periodicity, making it a promising candidate for electrocatalytic applications. We have analyzed AA and AB stacking model of TFPh_DHPh_COF which were presented along with their simulated PXRD pattern in Figure S4. Meanwhile, the XRD pattern of the control sample TFPh_DMePh_COF was also recorded in Figure S5, revealing its crystalline nature. The peak positions closely resembled those of TFPh_DHPh_COF, with the only difference being the substitution of two ─OH groups by two –Me groups. The Fourier transform infrared spectroscopy (FTIR) spectrum confirms the successful formation of the hydroxyl‐functionalized enamine‐ and imine‐linked COF (TFPh_DHPh_COF) by exhibiting characteristic vibrational bands corresponding to key functional groups (Figure 2b). Attenuation of sharp FTIR band at 1690 cm^−1^ (─C═O, from TFPh) along with the broad band at 3200 cm^−1^ (─NH_2_ from DHPhDA) and appearance of a strong band at 1607 cm^−1^ (─C═N─ from TFPh_DHPh_COF) provides reasonable evidence for the successful formation of the Schiff base linkage via polycondensation reaction. A broad absorption band observed around 3400 cm^−1^ is attributed to the ─O─H and ─N─H stretching vibration, indicating the presence of hydroxyl (─OH) and possible amine (─NH) functionalities.^[^ ^26^, ^28^ ^]^ The appearance of a sharp but low intense peak at 1677 cm^−1^ in the FTIR spectrum is attributed to the keto group, formed via β‐keto enamine tautomerization. The distinct peak at approximately 1607 cm^−1^ corresponds to C═N stretching, confirming the formation of imine linkages, which contribute to the active catalytic sites of the COF.^[^ ^29^ ^]^ Peaks within the 1200–1600 cm^−1^ range can be assigned to C─N and C═C stretching, indicative of an extended conjugated system that facilitates electron delocalization, crucial for electrocatalytic activity. Additionally, the fingerprint region below 1000 cm^−1^ shows characteristic bending and out‐of‐plane deformations associated with aromatic rings linkages, further supporting the ordered framework structure. These spectral features collectively validate the presence of essential functionalities within TFPh_DHPh_COF, reinforcing its structural integrity and potential applicability as a metal‐free electrocatalyst for ORR. The solid‐state NMR (MAS ^13^C‐NMR) spectrum confirms the successful formation of TFPh_DHPh_COF, displaying well‐resolved peaks corresponding to the characteristic chemical environments of the framework as shown in Figure 2c. The signals observed in the 150–160 ppm region are attributed to C═N (imine) and C─OH (phenolic) groups, validating the incorporation of key functional groups.

**Figure 3:**
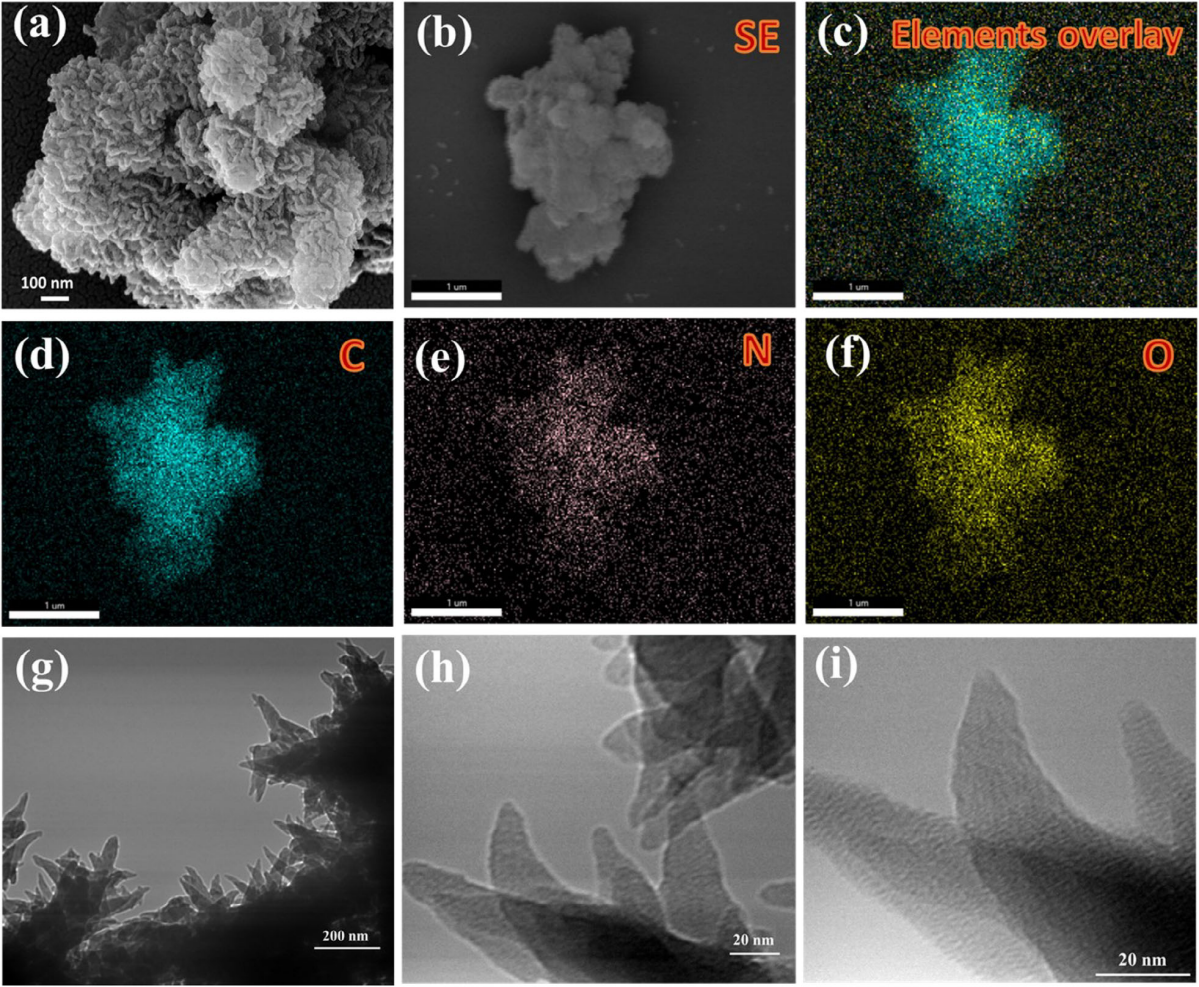
a) FESEM image of TFPh_DHPh_COF, elemental mapping of TFPh_DHPh_COF corresponding to b) electron image, c) elements overlay, d) carbon, e) nitrogen, and f) oxygen. g–i) Corresponding TEM images of as synthesized TFPh_DHPh_COF.

Additionally, a strong peak around 180 ppm suggests the presence of carbonyl group, formed via β‐keto enamine tautomerization.^[^ ^30^ ^]^ Peaks in the 120–140 ppm range correspond to aromatic carbon resonances, reflecting the π‐conjugated backbone essential for charge transport. The presence of C─N linkages is confirmed by peaks in the 90–110 ppm region, supporting the formation of enamine and imine functionalities.^[^ ^28^ ^]^ The molecular structure further illustrates the periodic arrangement of enamine, imine, and hydroxyl (─OH) functionalities, which enhance electronic delocalization, O_2_ adsorption, and catalytic stability. These structural characteristics confirm TFPh_DHPh_COF as a highly ordered, stable, and efficient metal‐free electrocatalyst for the ORR. Furthermore, FTIR spectrum (Figure S6) and solid state ^13^C‐NMR study (Figure S7) of as synthesized TFPh_DMePh_COF confirmed the bonding connectivity and formation of the extended polymeric material. The field emission scanning electron microscopy (FESEM) image provides a detailed morphological analysis of the synthesized TFPh_DHPh_COF material, revealing its distinct structural characteristics at the nanoscale. As shown in Figure 3a (Figure S8) the image shows an aggregated nanowire like morphology, indicating the formation of loosely packed interconnected nanostructures. The wrinkled and nanowire feature suggest a highly porous and hierarchical architecture, which is beneficial for electrocatalysis and mass transport in the ORR. The presence of rough and crumpled structures implies a high surface area, which enhances the accessibility of active sites and improves catalytic performance. The FESEM and elemental mapping analysis confirm the successful synthesis of TFPh_DHPh_COF, with uniform elemental distribution. The FESEM images (Figure 3b–f) highlight the elemental mapping which confirms the presence of carbon (C), nitrogen (N), and oxygen (O). The carbon framework (d) forms the organic backbone, nitrogen (e) validates enamine and imine linkages for charge delocalization, and oxygen (f) indicates hydroxyl (─OH) groups for enhanced stability and oxygen adsorption. These features confirm the well‐defined COF structure, reinforcing its potential as a metal‐free electrocatalyst for ORR, which is further validated by energy dispersive analysis of X‐rays (EDAX) spectra and atomic percentage (Figures S9 and S10). To further investigate the internal structure and morphology at the nanoscale, transmission electron microscopy (TEM) analysis was conducted. As shown in Figure 3(g–i), the high‐magnification TEM image reveals smilar nanostructured surface as that of FESEM analysis.

**Figure 4:**
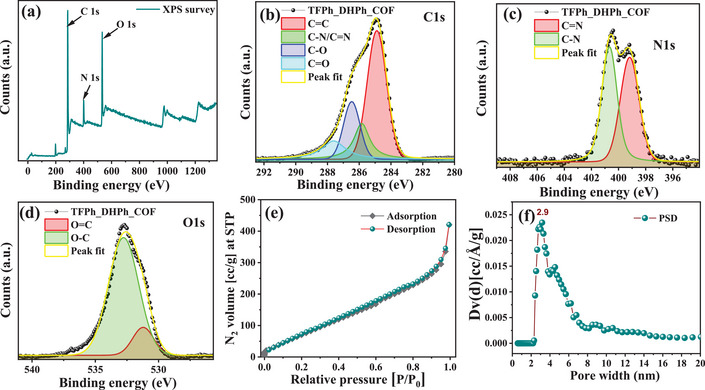
a) XPS survey of TFPh_DHPh_COF, containing C, N, and O. Deconvoluted spectrum of TFPh_DHPh_COF corresponding to b) C1s, c) N1s, and d) O1s. e) BET surface area of TFPh_DHPh_COF and f) pore‐size distribution of TFPh_DHPh_COF.

For the analysis of chemical environment of the TFPh_DHPh_COF, X‐ray photoelectron spectroscopy (XPS) analysis was performed. The XPS survey analysis confirmed the presence of all the key species such as carbon, nitrogen, and oxygen as shown in Figure 4a. The deconvoluted C1s spectrum provides insight into the nature of carbon‐bonding within the framework as depicted in Figure 4b. The spectrum reveals distinct peaks corresponding to different functional groups: the major peak at ∼284.8 eV is attributed to C═C bonds, indicative of sp^2^‐hybridized(c)(c) aromatic carbon, whereas the peak at ∼285.8 eV corresponds to C─N/C═N bonds, confirming the incorporation of imine (─C═N─) or enamine (─C─NH─) functionalities.^[^ ^31^ ^]^ Additionally, the presence of oxygen‐containing species is evidenced by peaks at ∼286.5 eV and ∼287.6 eV, assigned to C─O and C═O bonds, respectively, suggesting the presence of hydroxyl and carbonyl functionalities. Similarly, N1s analysis was conducted as shown in Figure 4c, and the deconvoluted spectrum reveals the presence of distinct nitrogen functionalities.^[^ ^32^ ^]^ The peak at approximately 399.5 eV is assigned to C═N bonds, indicative of imine (─C═N─) functionalities, which play a crucial role in the structural integrity and electronic properties of the COF. The second peak at ∼400.7 eV corresponds to C─N bonds, suggesting the presence of amine (─C─NH─) linkages. The presence of imine (─C═N─) and amine (─C─NH─) functionalities within the COF structure highlights its potential for electrocatalytic applications, as these nitrogen centers can modulate charge distribution and enhance catalytic activity.^[^ ^33^ ^]^ In order to determine the oxygen chemical environment in TFPh_DHPh_COF, O1s analysis was conducted (Figure 4d), and the deconvoluted spectrum reveals the presence of different oxygen species. The peak observed at ∼531.2 eV corresponds to O═C bonds, indicating the presence of carbonyl (─C═O) functionalities.^[^ ^34^ ^]^ The dominant peak at ∼532.9 eV is assigned to O─C bonds, signifying the presence of hydroxyl (─OH) group. The peak fitting (yellow line in each plot) demonstrates a well‐resolved deconvolution, confirming the distinct contributions of functional groups.

In order to determine the surface area and porosity of TFPh_DHPh_COF, N_2_ adsorption–desorption isotherm and pore size distribution analysis was conducted. Figure 4e presents the N_2_ adsorption–desorption isotherm of TFPh_DHPh_COF at 77 K, which follows a characteristic type I followed by type IV isotherm with a slight hysteresis loop indicating the presence of mesopores in extended structures. The BET surface area was calculated to be 388 m^2^ g^−1^, confirming the moderately high porosity of the material, which is essential for facilitating mass transport and active site accessibility in electrocatalysis. Figure 4f shows the pore size distribution, obtained from the N_2_ adsorption–desorption isotherm using the nonlocal density functional theory (NLDFT) method, revealing a dominant pore size of approximately 2.9 nm, confirming the mesoporous nature of TFPh_DHPh_COF.^[^ ^35^ ^]^ In the isotherm a steady increase of N_2_ adsorption with the increase of pressure, infer the possibility of deformation/swelling of porous network which could be validated through the unsymmetrical‐bonding linkage (both amine, imine) presence in COF structure. Sharp increase of N_2_ absorption at the high‐pressure region was also indicated the interparticle porosity of the nano structure. This moderately high surface area and mesoporous 2D channel along π–π stacked COF structure features the facile ions transportation as well as gives the opportunity to accessible catalytic sites for the easy interaction with O_2_ molecules to be reduced.

**Figure 5:**
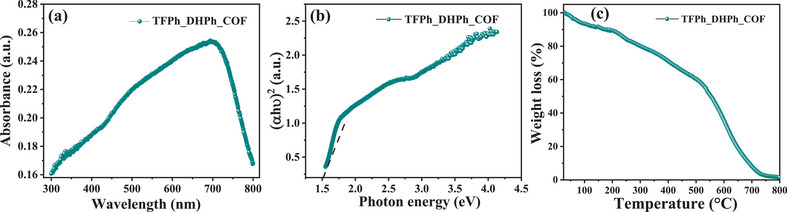
a) UV–vis absorption spectrum of TFPh_DHPh_COF. b) Tauc Plot for the estimation of the optical band gap of TFPh_DHPh_COF. c) Thermogravimetric analysis of TFPh_DHPh_COF.

To investigate the optical properties and electronic band structure of TFPh_DHPh_COF, UV–vis absorption spectroscopy and Tauc plot analysis were performed. Figure 5a illustrates the absorbance spectrum, the strong absorption in the visible region indicates that TFPh_DHPh_COF has an extended π‐conjugated system, enhancing its electronic delocalization and charge transfer properties which directly corelates with the band gap of the as synthesized COF. Tauc plot, which is used to extract the optical band gap of TFPh_DHPh_COF, which was calculated to be ∼1.5 eV (Figure 5b). From computational study, the most favorable complexation site displayed the lowest HOMO–LUMO gap of 1.641 eV which is very close to the band gap of the sole COF material. The estimated band gap value, together with the presence of nitrogen (C═N, C─N) and oxygen (C═O, C─O) functionalities, suggests that TFPh_DHPh_COF exhibits a tuneable electronic structure, making it a promising candidate for electrocatalytic applications, such as the ORR. Thermal stability analysis is very crucial for fixing the outgassing temperature for the study of BET surface area analysis. In this purpose TGA analysis has been done under air (Figure 5c). The nano porous structure is thermally stable until 480°C. 1^st^ break of surface adsorb moisture, then up to 200°C might be for functional group like ─OH then beyond 480°C the COF structure has been collapsed.

### 2.4 Electrochemical ORR Study

**Figure 6:**
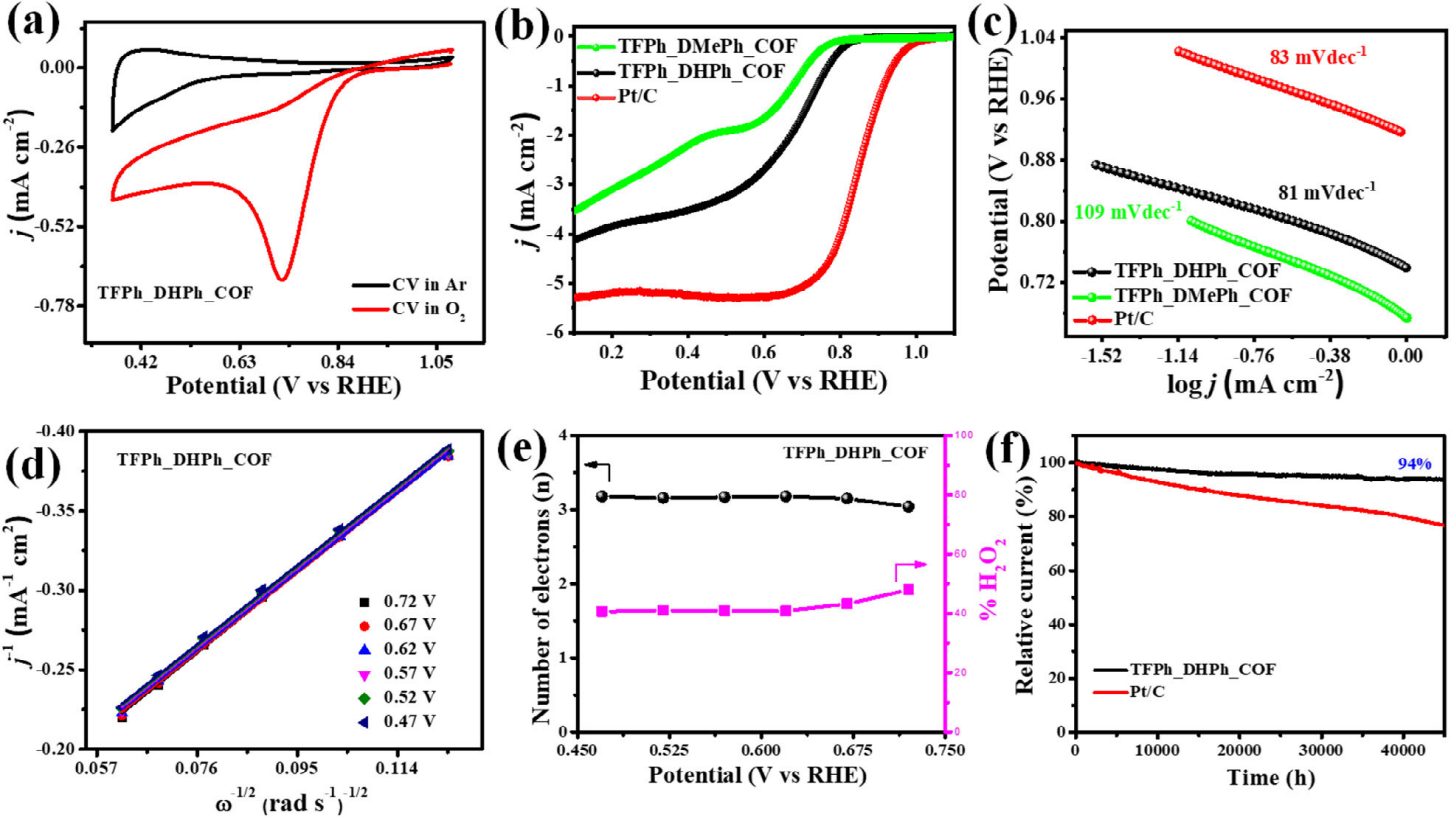
a) CV of TFPh_DHPh_COF in argon and oxygen‐saturated 0.1 M KOH solution. b) LSV curves of as synthesized TFPh_DHPh_COF, TFPh_DMePh_COF, and Pt/C catalyst in O_2_‐saturated alkaline electrolyte solution. c) Tafel plot of catalyst; comparison with TFPh_DMePh_COF and Pt/C catalyst. d) K–L plots of TFPh_DHPh_COF catalyst, e) number of electrons involved, and %H_2_O_2_ yield formed during ORR with TFPh_DHPh_COF catalyst in alkaline solution. f) Stability curves of TFPh_DHPh_COF and Pt/C catalysts.

The electrocatalytic performance of TFPh_DHPh_COF was evaluated using cyclic voltammetry (CV) measurements in a 0.1 M KOH solution, recorded at a scan rate of 10 mV s^−1^. The presence of a distinct cathodic peak in an O_2_‐saturated electrolyte confirms the catalytic activity for ORR of the as synthesized catalyst, which was absent in an Ar‐saturated environment, demonstrating its selective interaction with molecular oxygen (Figure 6a). To further investigate the ORR kinetics, linear sweep voltammetry (LSV) was conducted using a rotating ring‐disk electrode (RRDE) at 1600 rpm, revealing a half‐wave potential (E_1/2_) of 0.72 V vs RHE which is 150 mV negative to those of commercial Pt/C (20 wt %) and a limiting current density (J_I_) of 4.2 mA cm^−2^, as shown in Figure 6b. A comparative summary of the electrocatalytic performance toward ORR of recently reported COF‐based catalysts is presented in Table S2. The Tafel slope, derived from polarization curves, was calculated to be 81 mV dec^−1^ for TFPh_DHPh_COF material and 83 mV dec^−1^ for commercial Pt/C (Figure 6c), highlighting the catalyst efficient faster electron transfer and favorable ORR reaction kinetics. Role of ─OH functionality toward ORR was also investigated with another similar COF (TFPh_DMePh_COF) where two ─OH groups were substituted by two ‐Me groups. In this case the obtained LSV and Tafel slope were 0.68 V and 109 mVdec^−1^, respectively. The poor electrochemical ORR performance by ‐Me substituted COF might be responsible for less electron donating properties of ‐Me group with respect to ─OH group which further justifies the role of ─OH linkage in the as synthesized COF. The electrochemical active surface area (ECSA) was determined through CV scans at varying scan rates (Figure S11a,b), yielding an ECSA value of 9.45 cm^2^, indicating a high density of accessible catalytic active sites. Additionally, electrochemical impedance spectroscopy (EIS) analysis revealed a lower charge transfer resistance (R_ct_) at the electrode/electrolyte interface, signifying enhanced conductivity and rapid charge transport, as evident from the Nyquist plot (Figure S12a,b). The ORR mechanism was further explored by performing LSV at different rotation speeds (625–2500 rpm), where the increase in limiting current density with higher rotation speeds confirmed improved oxygen diffusion kinetics (Figure S13). The Koutecky–Levich (K–L) plots exhibited linearity at various potentials 0.47 V to 0.72 V vs RHE (see Figure 6d), suggesting that the catalyst follows first‐order ORR reaction kinetics with respect to dissolved oxygen concentration.^[^ ^36^, ^37^ ^]^To gain further insight into the reaction pathway, the RRDE technique was used to determine the electron transfer number (*n*) and peroxide intermediate formation by using LSV at 1600 rpm (Figure S14). The calculated *n*‐value ranged from 3.07 to 3.19 (Figure 6e), indicating that ORR predominantly follows a four‐electron reduction pathway with TFPh_DHPh_COF catalyst material, leading to the direct conversion of O_2_ into H_2_O while minimizing the generation of H_2_O_2_ by‐products. The H_2_O_2_ yield remained below 42%, further verifying the selectivity of catalyst toward the 4e⁻ pathway. Stability assessments conducted through chronoamperometric measurements at 0.57 V vs RHE demonstrated 94% current retention after 12 h, significantly outperforming commercial Pt/C (20 wt %) catalyst material, which suffered from degradation due to metal leaching in alkaline conditions (Figure 6f). The synthesized TFPh_DHPh_COF catalyst material shows high durability because of abundant active sites available for the ORR. These findings establish TFPh_DHPh_COF as a highly stable, selective, and metal‐free electrocatalyst with promising potential for fuel cell and metal–air battery applications.

### 2.5 Mechanistic Insights and Active Site Interactions for ORR: DFT Approach

**Figure 7:**
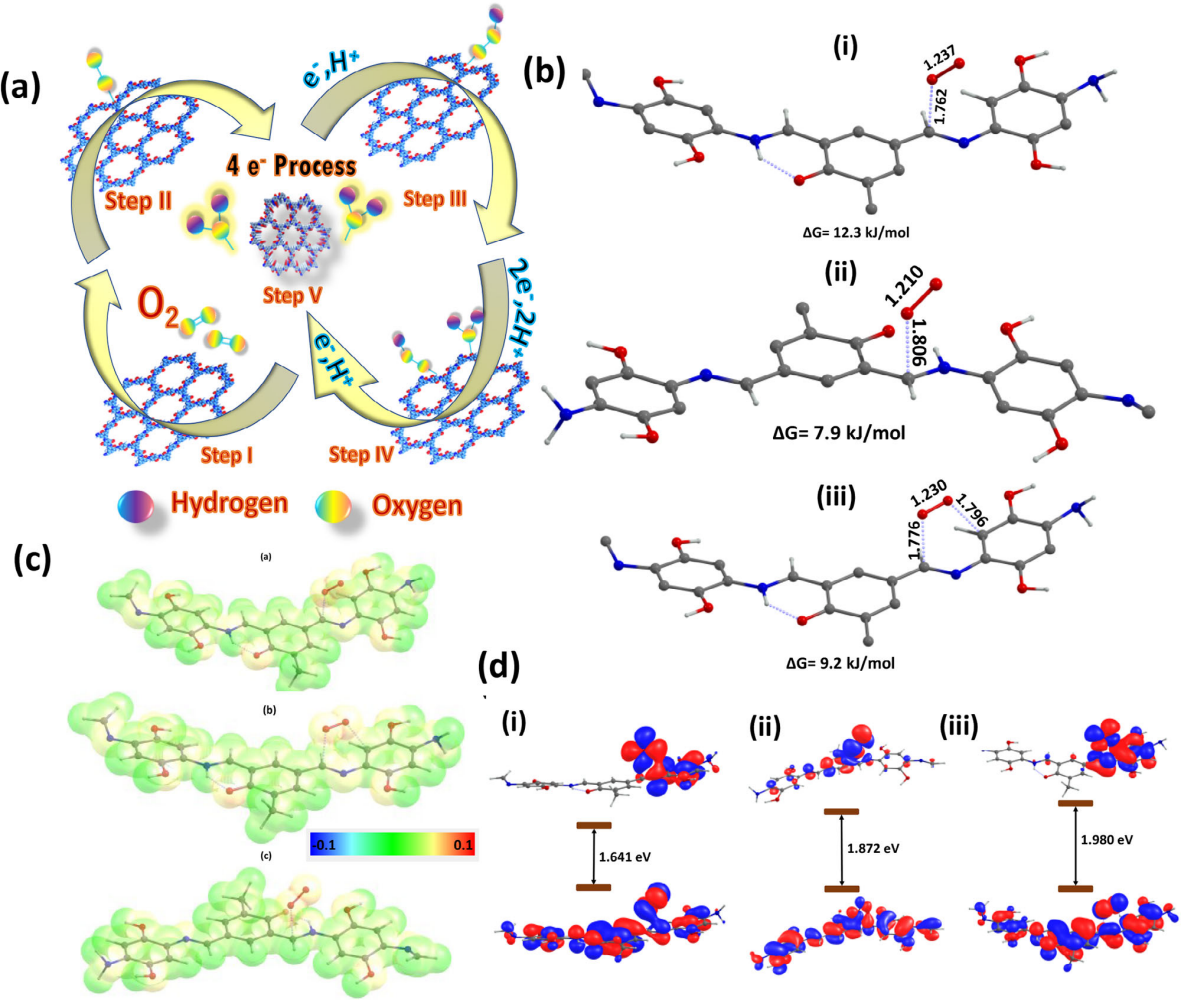
a) Mechanistic 4e^−^ path of the ORR on the catalyst surface. b) TFPh_DHPh_COF with dual binding sites interact with O_2_, optimized at B3LYP/6‐31** level of theory. The bond length of the adsorbed O_2_ is mentioned in Å for each complex, showing that O_2_ bond length in these complexes has increased compared to that of the pure O_2_. c) Charge distribution curve of the TFPh_DHPh_COF catalyst at 0.004 iso‐surface value on atoms. d) HOMO–LUMO gap of the TFPh_DHPh_COF catalysts with dual binding sites interacting with O_2_, plotted with an isosurface value of 0.01e^−^ per bohr^3^ (hydrogens are omitted for clarity; color code: red: oxygen, blue; nitrogen, and dark grey: carbon atoms).

In this study, DFT calculations were employed to investigate the mechanistic aspects of ORR in TFPh_DHPh_COF matrial, with a particular focus on weak interactions between COFs and O_2_ molecules. Two distinct functional groups, enamine and imine, were identified as potential active sites facilitating O_2_ adsorption and activation. The interaction between these active sites and molecular oxygen was analyzed through two distinct binding models, including the Yeager and Pauling models. Among these configurations, the Pauling model at the imine site exhibited the highest binding free energy, indicating its superior efficiency in O_2_ binding with free energy of complexation 12.3 kJ/mol. The catalytic cycle, including intermediate formation during the ORR process, is schematically illustrated in Figure 7a. Upon O_2_ adsorption, a noticeable elongation of the O─O bond to 1.237 Å was observed, the longest among all binding configurations, further Supporting the activation of molecular oxygen (Figure 7b(i–iii)). Additionally, natural bond orbital (NBO) calculations revealed a partial positive charge (*C* ^δ⁺^ = 0.12) on the carbon atom participating in O_2_ adsorption, consistent with the end on binding mode described in the Pauling model (Figure 7c(i–iii)).^[^ ^38^ ^]^ To further elucidate the reaction mechanism, a molecular orbital (MO) analysis indicate that energy gap between the highest occupied molecular orbital (HOMO) and the lowest unoccupied molecular orbital (LUMO) was computed for each potential binding site (Figure 7d(i–iii)). The most favorable complexation site displayed the lowest HOMO–LUMO gap of 1.641 eV, signifying enhanced electron transfer characteristics and interaction stability.

These findings provide strong evidence for the high catalytic efficiency of the Pauling model) at the imine site in facilitating ORR, primarily through weak yet significant interactions that enhance O_2_ activation.^[^ ^39^ ^]^ Moreover, in the extended electronic structure of TFPh_DHPh_COF both ─C═N and ─C─NH linkage are present where imine functionality is responsible for ORR and amine linkage providing the structural stability through the hydrogen‐bonding interaction with oxygen atom. Extended π‐electron delocalization through the whole 2D COF structure and this kind of hydrogen‐bonding interaction, enhanched its long duration electrochemical stability over Pt/C. In addition, to investigate the specific contribution of the hydroxyl (─OH) group to the O_2_ adsorption process in the COF framework, we performed a functional group substitution by replacing the ─OH groups with two methyl (─CH_3_) group, which lacks comparable electron‐donating capability (Figures S15 and S16). Comparative Gibbs free energy calculations for O_2_ binding revealed that the methyl‐substituted COF exhibits a higher Gibbs free energy (i.e., less favorable adsorption) relative to the hydroxyl‐functionalized system. This decrease in binding affinity upon ─OH substitution strongly suggests that the hydroxyl group plays a critical role in stabilizing the adsorbed O₂ molecule, likely through electron donation or hydrogen‐bonding interactions. These findings highlight the significance of functional group selection in modulating O_2_ adsorption properties on catalyst surfaces and enhancing the ORR.

## 3 Conclusion

In this study, we have successfully synthesized and characterized a hydroxyl‐functionalized enamine‐ and imine‐linked COF (TFPh_DHPh_COF) material as an efficient metal‐free electrocatalyst for the ORR in alkaline media. The high surface area, hierarchical porosity, and well‐defined π‐conjugated structure enabled superior charge transport and oxygen adsorption, leading to enhanced electrocatalytic activity. Electrochemical evaluations demonstrated a half‐wave potential (0.72 V vs. RHE), and a low Tafel slope (81 mV dec^−1^), high durability, highlighting the excellent kinetics and ORR efficiency toward metal free catalyst material. The chemistry of active sites via Pauling and Yeager models from theoretical aproach has been explored. The >C═N‐imine linkage in the COF materials serves as an active site for O_2_ activation by end on binding and further reduction, stabilizes important intermediates, and encourages a favorable reaction pathway leading to the four‐electron ORR process. Additionally, the β‐keto enamine linkage of the COF structure furnished extra structural stability. The findings of this study establish TFPh_DHPh_COF material as a promising alternative to noble metal or transition metal catalysts, offering a scalable, cost‐effective, and sustainable solution for fuel cell and metal–air battery applications in alakline media.

## Supporting Information

The authors have cited additional references within the Supporting Information.^[^ ^17^, ^40^, ^41^, ^42^, ^43^, ^44^, ^45^, ^46^, ^47^, ^48^, ^49^, ^50^, ^51^ ^]^

## Conflict of Interests

The authors declare no conflict of interest.

## Supporting information



Supporting Information

## Data Availability

The data that support the findings of this study are available from the corresponding author upon reasonable request.
