# Supplementary material for: Polyhydroxy‐Decorated 2D Covalent Organic Framework with Imine Active Sites toward Efficient Electrocatalytic Oxygen Reduction Reaction: Experimental and Theoretical Insights
Source: Chem Asian J. 2025 Jul 8;20(18):e00592. doi: 10.1002/asia.202500592 (PMC12450041; doi:10.1002/asia.202500592)
Supplement: Supplementary file 1 — Supporting Information [file ASIA-20-e00592-s001.docx]

**Supporting Information**

**Polyhydroxy-Decorated 2D Covalent Organic Framework with Imine Active Sites Towards Efficient Electrocatalytic Oxygen Reduction Reaction: Experimental and Theoretical Insights**

Manisha Das, ^[a]^ Thakur Rochak Kumar Rana, ^[b]^ Santanu Chand, ^[c]^ Sumit Kumar, ^[d]^ Takaya Ogawa,^[a]^ Laurent Billon, ^[e]^ Tokuhisa Kawawaki,^[f]^ Sabuj Kanti Das,*^[e,f,g]^ and Yuichi Negishi*^[f,g]^

[a] Prof. T. Ogawa, Dr. M. Das

Graduate School of Energy Science, Kyoto University, Yoshida-honmachi, Sakyo-ku, Kyoto 606-8501, Japan

[b] T. R. K. Rana

Department of Chemistry, Indian Institute of Technology, Bombay, Powai, Mumbai-400076, India

[c] Dr. S. Chand

Department of Applied Chemistry, Graduate School of Engineering, The University of Tokyo, Bunkyo-ku, Tokyo 113-8656, Japan

[d] S. Kumar

Department of Physics, N.R.E.C. College Khurja, 203131 Uttar Pradesh, India

[e] Dr. S. K. Das* and Prof. L. Billon

Bio-Inspired Materials Group: Functionalities & Self-Assembly, Universite de Pau et des Pays de l'Adour, E2S UPPA, UPPA/CNRS, IPREM UMR 5254, 2, Avenue du Président Angot, 64053 Pau, France

[f] Dr. S. K. Das*, Dr. T. Kawawaki, Prof. Y. Negishi*

Institute of Multidisciplinary Research for Advanced Materials, Tohoku University, Aoba-ku, Sendai 980-8577, Japan

[g] Dr. S. K. Das*, Prof. Y. Negishi*

Research Institute for Science & Technology, Tokyo University of Science, Tokyo 162-8601, Japan

^⁎^Corresponding Authors

E-mail: sabujkanti.iitb@gmail.com

E-mail: [yuichi.negishi.a8@tohoku.ac.jp](mailto:yuichi.negishi.a8@tohoku.ac.jp)

- 1. **Instrumentation**

Powder X-ray diffraction (PXRD) analysis was conducted using Rigaku miniFlex600 with a Cu-Kα radiation source (λ = 1.5406 Å), operating within a 2θ range of 3° to 70° at an acceleration voltage of 40 kV to examine the crystal structure of the samples. Solid-state ¹³C NMR spectroscopy was performed using an NMR600 instrument at SAIF, IIT Bombay, with structural confirmation carried out using a JEOL NMR600 spectrometer. The Field Emission Scanning Electron Microscope (FESEM) was utilized for detailed surface morphology characterization. Transmission electron microscopy (TEM) was performed using a HITACHI 87650 instrument to analyse the detailed morphology of the synthesized COF. For surface area determination, N₂ sorption analysis was performed at 77 K using an Quantachrome autosorbiQ-MP-MP-MP (3 Stat.) gas adsorption analyser, with COF samples degassed at 150°C for 3 hours before measurement. The specific surface area and pore size distribution were analysed using the Brunauer-Emmett-Teller (BET) method and the Non-Local Density Functional Theory (NLDFT) model, respectively. To evaluate the bonding configuration and elemental composition, X-ray photoelectron spectroscopy (XPS) was carried out using a K-Alpha 1063 spectrometer in an ultrahigh vacuum chamber (7×10⁻⁹ torr). All spectral measurements were conducted under ambient conditions, which were consistently maintained throughout the experiment. For the study of electrochemical measurements, Simadzu-RRDE-3A instruments were utilized. To elucidate the structure of the COFs and determine the unit cell parameters, potential 2D models were optimized using Gaussian-09 and Materials Studio. Various stacking configurations were explored based on previously reported literature. The experimental PXRD patterns exhibited good agreement with the simulated patterns of near-eclipsed stacking models. (Accelrys, Material Studio Release Notes, Release 4.2, Accelrys Software, San Diego (2006).

**1.2. Chemicals**

Various chemical reagents, including resorcinol (98%), hexamine (99%), 2,5-diaminobenzene-1,4-diol. dihydrochloride (DHPhDA), hydrochloric acid (HCl, 98%), trifluoroacetic acid, toluene, ethanol, N, N-dimethylformamide (DMF), and 1,4-dioxane, were sourced from Sigma-Aldrich, Merck, and TCI. 2,5-Dimethyl-1,4-phenylenediamine (DMePhDA) was received from TCI, Japan. These chemicals, along with all solvents, acids, and bases, were employed directly as received, without undergoing any further purification processes.

**1.3.** **Synthesis of 2,4,6-Triformylphenol (TFPh)**

Following an adapted protocol, a mixture of 7.16 g of purified phenol and 20 g of hexamine was placed in a 100 ml round-bottom flask and cooled in an ice bath. Subsequently, 65 mL of trifluoroacetic acid was introduced, and the reaction was maintained at 120°C for 24 hours. The temperature was then increased to 150°C for an additional 3 hours under a nitrogen (N₂) atmosphere. Upon completion, the reaction mixture was gradually cooled to 120°C before the addition of 110 mL of 3M HCl, followed by stirring at 105°C for 30 minutes. The solid product that precipitated was hot-filtered, washed thoroughly with 100 mL of ethanol (EtOH), and recrystallized from hot N, N-dimethylformamide (DMF), yielding 2,4,6- Triformylphenol with a final yield of 30%. The purified compound was subsequently analyzed using ¹H NMR spectroscopy.^[1]^

**1.4. Synthesis of TFPh_DMePh_COF**

For the synthesis of TFPh_DMePh_COF, a molar ratio of 2:3 of TFPhe and DMePhDam were taken in a in a Schlenk tube, followed by the addition of a catalytic amount of acetic acid (AcOH) and a solvent mixture of dioxane and mesitylene. The reaction mixture was degassed using the freeze–pump–thaw method, and the closed tube was then heated in an oil bath at 110 °C for 3 days to yield the TFPh_DMePh_COF powder. Subsequently, the product was purified by successive washings with DMF, THF, and ethanol (EtOH), dried under vacuum, and the final material was collected for further study.

**1.5. Electrode fabrication**

To ensure a clean and well-prepared electrode surface, the glassy carbon (GC), rotating disk electrode (RDE), and rotating ring-disk electrode (RRDE) underwent sequential polishing with 1.0, 0.3, and 0.05 μm alumina (Al_2_O_3_) powder, followed by ultrasonic treatment in deionized (DI) water to eliminate any residual contaminants. The catalyst suspension was formulated by dispersing the COF catalyst powder and acetylene black activated carbon (1:1) in a 50:50 (v/v) mixture of DI water and isopropyl alcohol (IPA), followed by 30 minutes of sonication to achieve a homogenous dispersion. This well-mixed catalyst ink was then drop-cast onto the electrode surface, ensuring an optimized loading of 0.8 mg cm^-2^. For reference, Pt/C (20 wt%) and RuO₂ electrodes were prepared by suspending Pt/C in a carefully controlled mixture of Milli-Q water, ethanol, and 5% Nafion, ensuring uniformity before application.

**1.6. Determination of Electron Transfer Number and H₂O₂ Yield**

To analyze the oxygen reduction reaction (ORR) mechanism, the number of transferred electrons (n) and the percentage of hydrogen peroxide (H₂O₂ yield) were determined through RRDE experiments, applying the following equations:

$n=4\times\frac{I\text{D}}{I\text{D}+ \frac{I\text{R}}{N}}$

(3)

(2)

$$\%H\text{2}O\text{2}\text{= 200× }\frac{\frac{I\text{R}}{N}}{\frac{I\text{R}}{N}+I\text{D}}$$

where *I*_D_ and *I*_R_ represent the disk and ring currents, respectively, and **N** denotes the Pt ring collection efficiency, which in this case is 0.249.

**1.7. Kinetic Analysis Using the Koutecky-Levich (K-L) Equation**

The kinetic current density (J_K_) was determined using the Koutecky-Levich (K-L) equation to assess the ORR kinetics:

$\frac{\boldsymbol{1}}{\boldsymbol{J}}=\frac{\boldsymbol{1}}{\boldsymbol{J}\text{L}}+\frac{\boldsymbol{1}}{\boldsymbol{J}\text{K}}$=$\frac{\boldsymbol{1}}{\boldsymbol{B}\boldsymbol{\omega}^{\frac{\boldsymbol{1}}{\boldsymbol{2}}}}+\frac{\boldsymbol{1}}{\boldsymbol{J}\text{K}}$ (4)

$B$=$0.62nFC\text{0 }{D\text{0}}^{2/3}\vartheta^{-1/6}$ (5)

$J\text{K}\text{ = nFk}\text{C}\text{0}$ (6)

In the equations above, J refers to the overall current density, J_L_ and J_K_ represent the diffusion-limited and kinetic current densities, respectively, and ω is the angular velocity of the rotating electrode (ω = 2πN, where N is the rotation speed). The constants include Faraday’s constant (F = 96485 C mol^-1^), oxygen bulk concentration (C₀), kinematic viscosity of the electrolyte (ν), and the diffusion coefficient of O_2_ in 0.1 M KOH (D₀ = 1.9 × 10^-5^ cm^2^ s^-1^). The n-value and kinetic current density (J_K_) were determined from the slope and intercept of the K-L plots.

**1.8. Oxygen Reduction Reaction (ORR) Mechanism**

The ORR, occurring at the cathode in fuel cells and metal-air batteries, follows a sequence of fundamental steps involving oxygen adsorption, electron transfer, and bond cleavage. The primary reaction steps include:

1. Oxygen diffusion and adsorption onto the electrode surface.
2. Electron transfer from the anode to the adsorbed O_2_ molecules.
3. Breaking of the O=O bond to generate oxygen intermediates.
4. Formation and removal of OH⁻ ions into the electrolyte solution.

ORR can proceed via either a direct four-electron pathway or a two-step two-electron pathway, with the former being more efficient:

$$O_{2}+ H_{2}O+{4e}^{-}\to{4OH}^{-}(0.401 V vs SHE)$$

$$O_{2}+ {H^{+}+4e}^{-}\to H_{2}O (1.229 V vs SHE)$$

where SHE refers to the standard hydrogen electrode. The four-electron pathway is preferred due to its higher efficiency and lower overpotential, whereas the two-electron pathway generates hydrogen peroxide (H_2_O_2_) as an intermediate, making it less desirable for applications requiring complete oxygen reduction.

**1.9. Computational Details**

**Methodology:** In this study, all Density Functional Theory (DFT) calculations were performed using the Gaussian16 Rev B.01 quantum chemical software package.3 Geometry optimizations were carried out employing the Grimme's dispersion-corrected B3LYP functional (B3LYP-D3).4,5 To describe the atomic species, the 6-31G** basis set was used for carbon, hydrogen, nitrogen, and oxygen atoms. Frequency calculations were performed to confirm the nature of the stationary points on the potential energy surface (PES) and to provide thermal corrections to the free energy.6,7 The optimized geometries were visualized using Chemcraft (version 1.6). Further analysis, including Natural Bond Orbital (NBO) calculations, was conducted at the B3LYP/Def2-TZVP level of theory, incorporating Grimme’s GD3 dispersion corrections. The NBO analysis provided insights into the electronic structure and bonding characteristics.

**Figure S1.** Synthesis of 2,4,6-Triformylphenol (TFPh)

**Figure S2.** Tautomerism of TFPh_DHPh_COF.


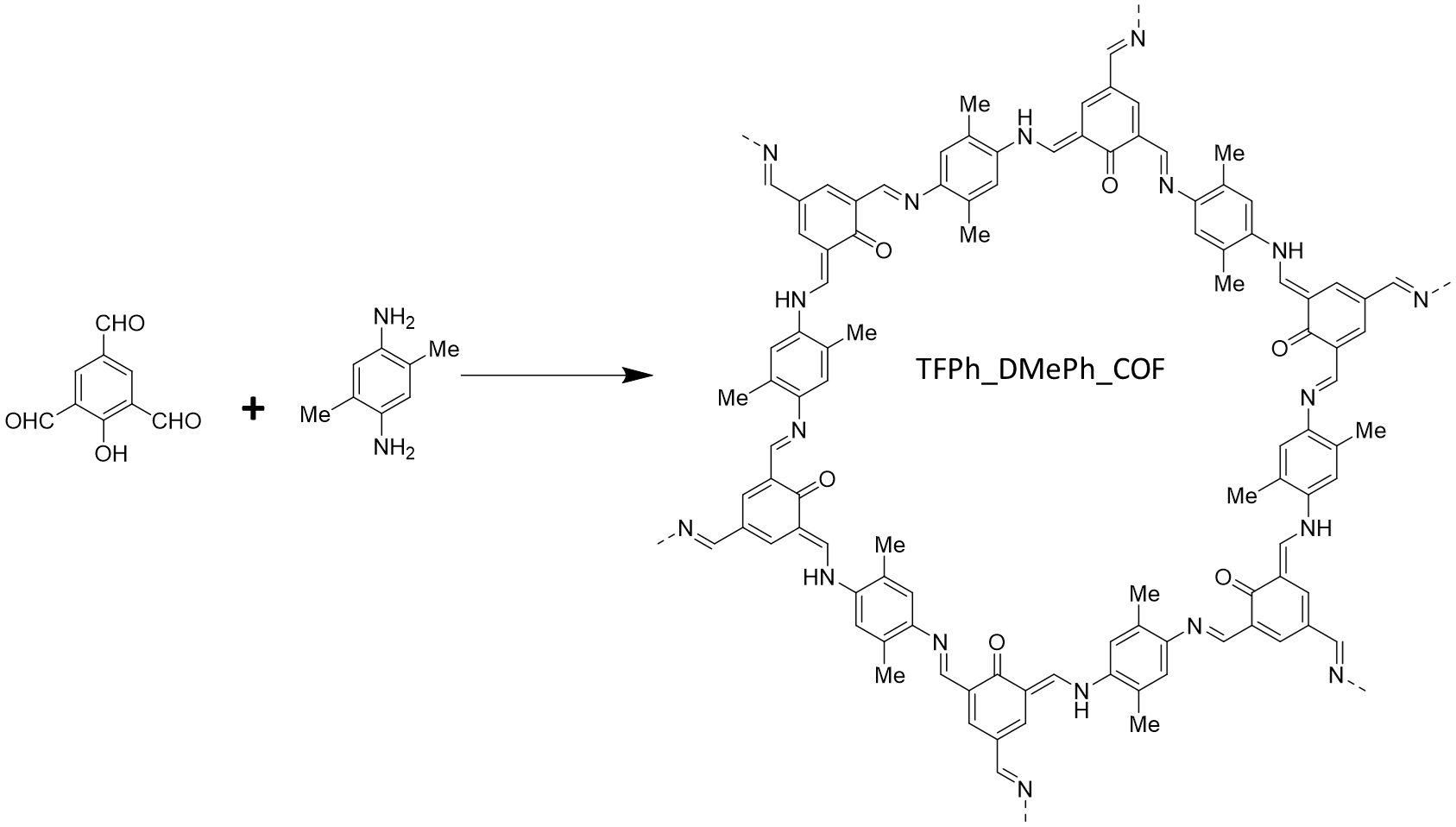


**Figure S3.** Synthesis of TFPh_DMePh_COF.

**Figure S4.** Simulated PXRD and AA and AB stacking structure of TFPh_DHPh_COF
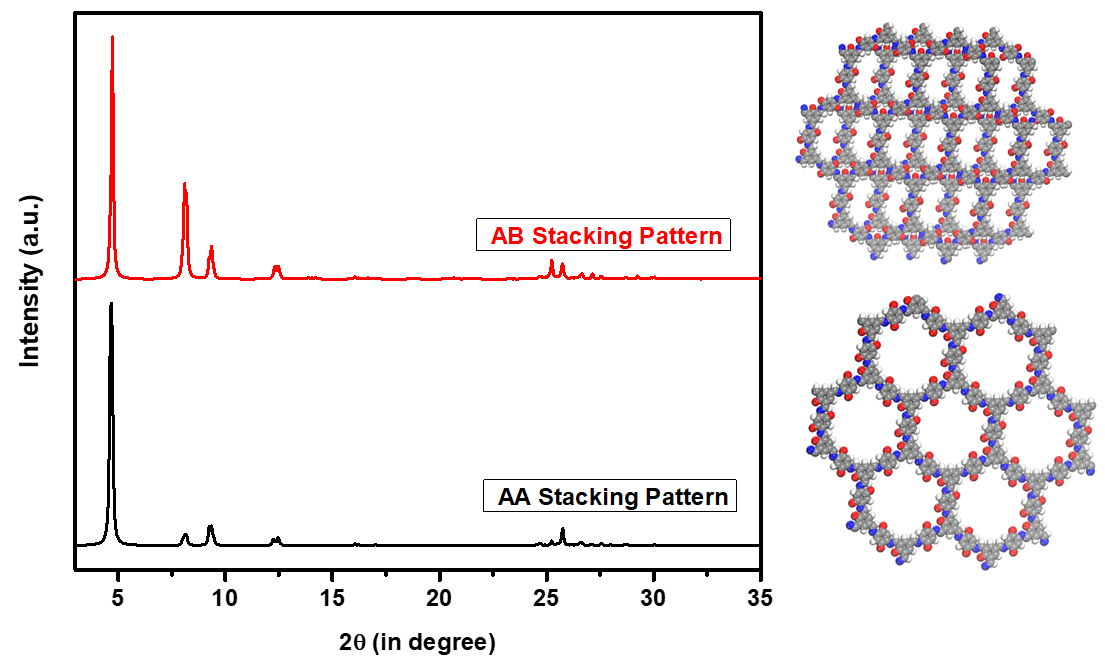
.

 **Figure S5.** XRD pattern of TFPh_DMePh_COF.


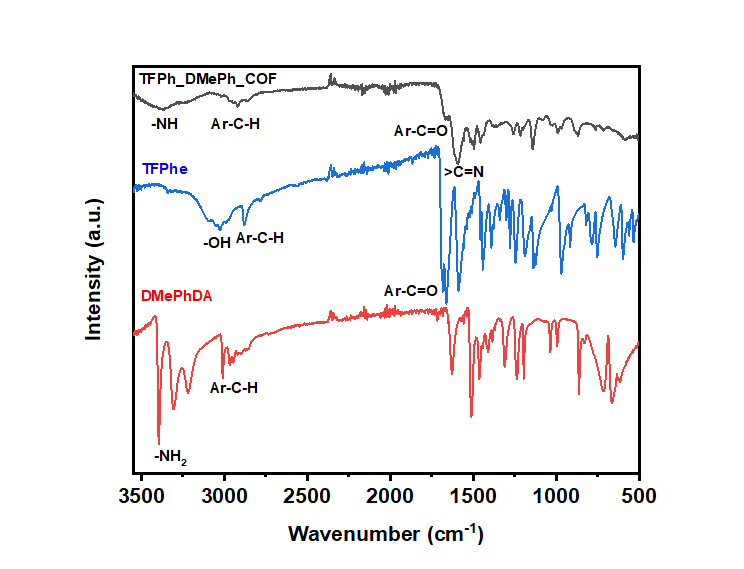
 **Figure S6.** FTIR spectra of TFPh_DMePh_COF, TFPhe and TFPh_DMePh_COF.


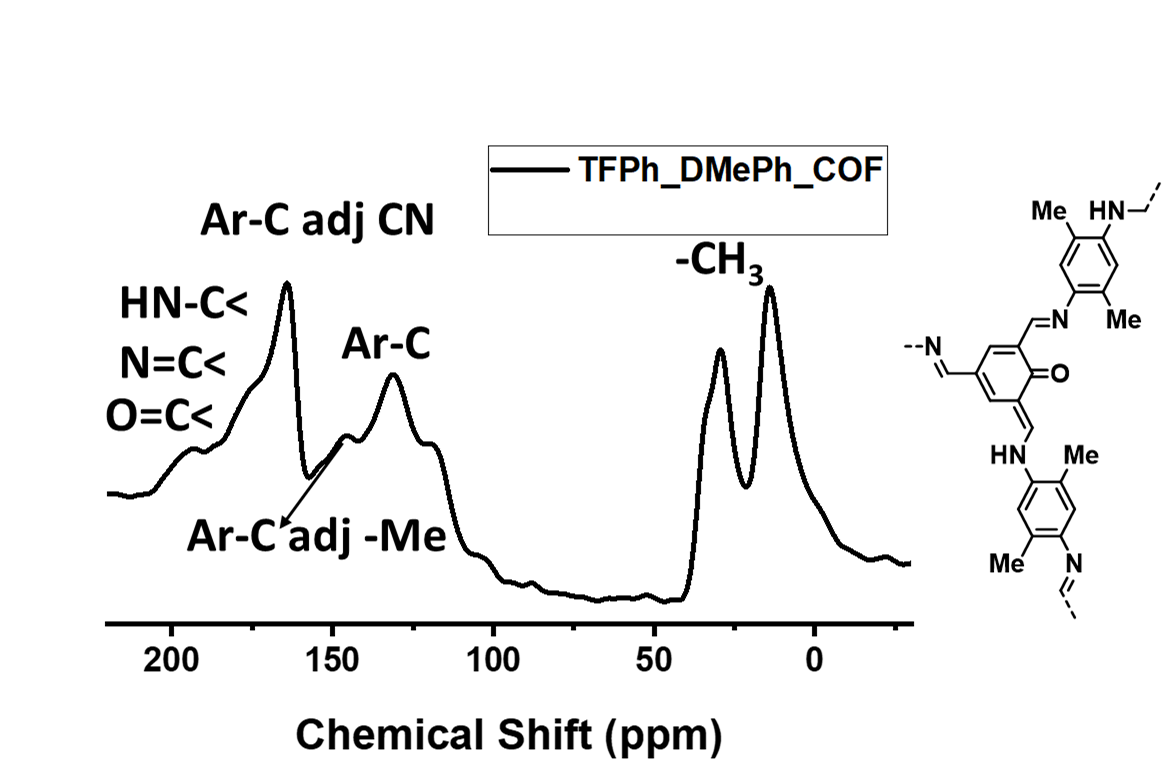


**Figure S7.** Solid-state NMR (MAS ^13^C-NMR) spectrum of TFPh_DMePh_COF.


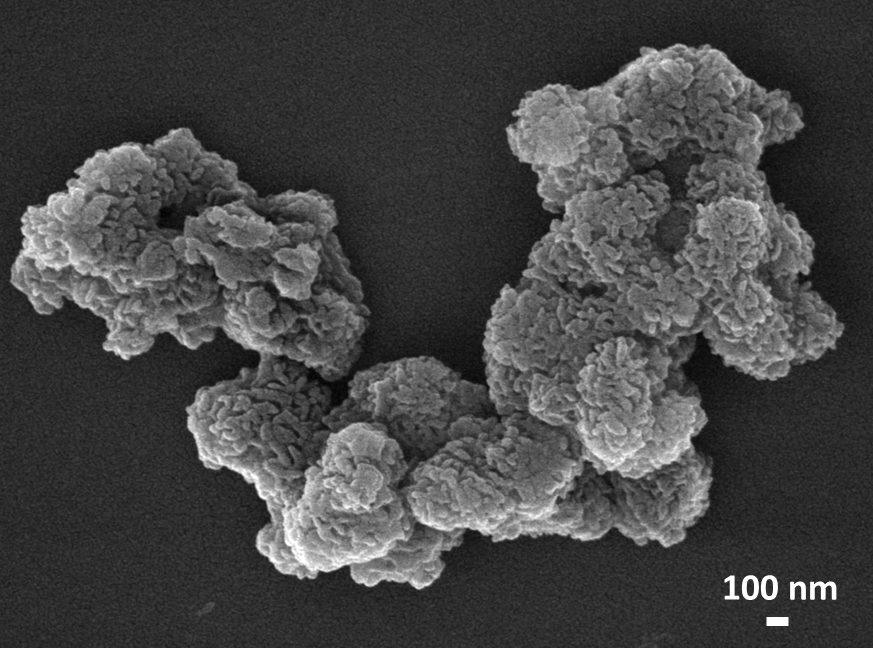


**Figure S8.** FESEM image of TFPh_DHPh_COF.


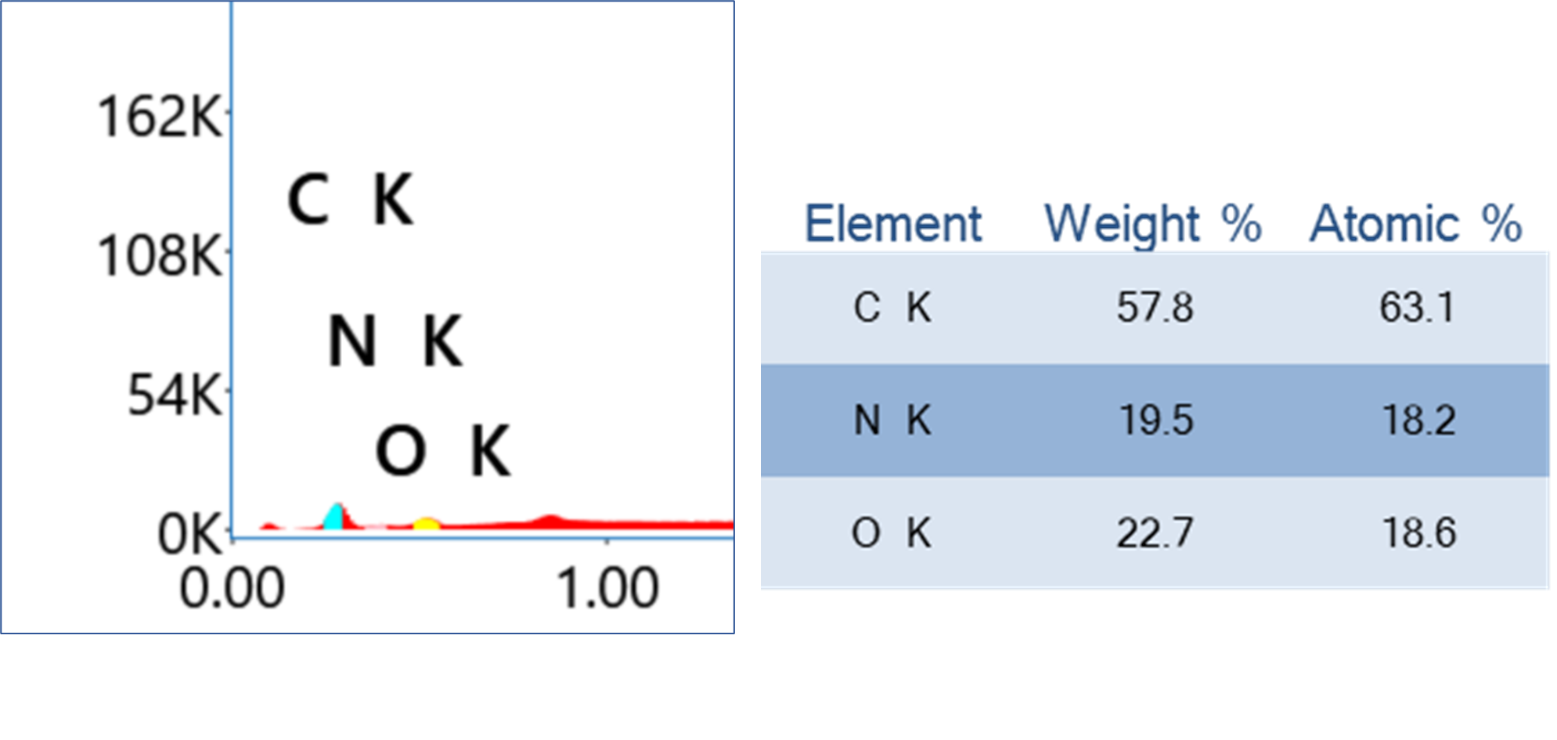


**Figure S9.** EDX of TFPh_DHPh_COF.


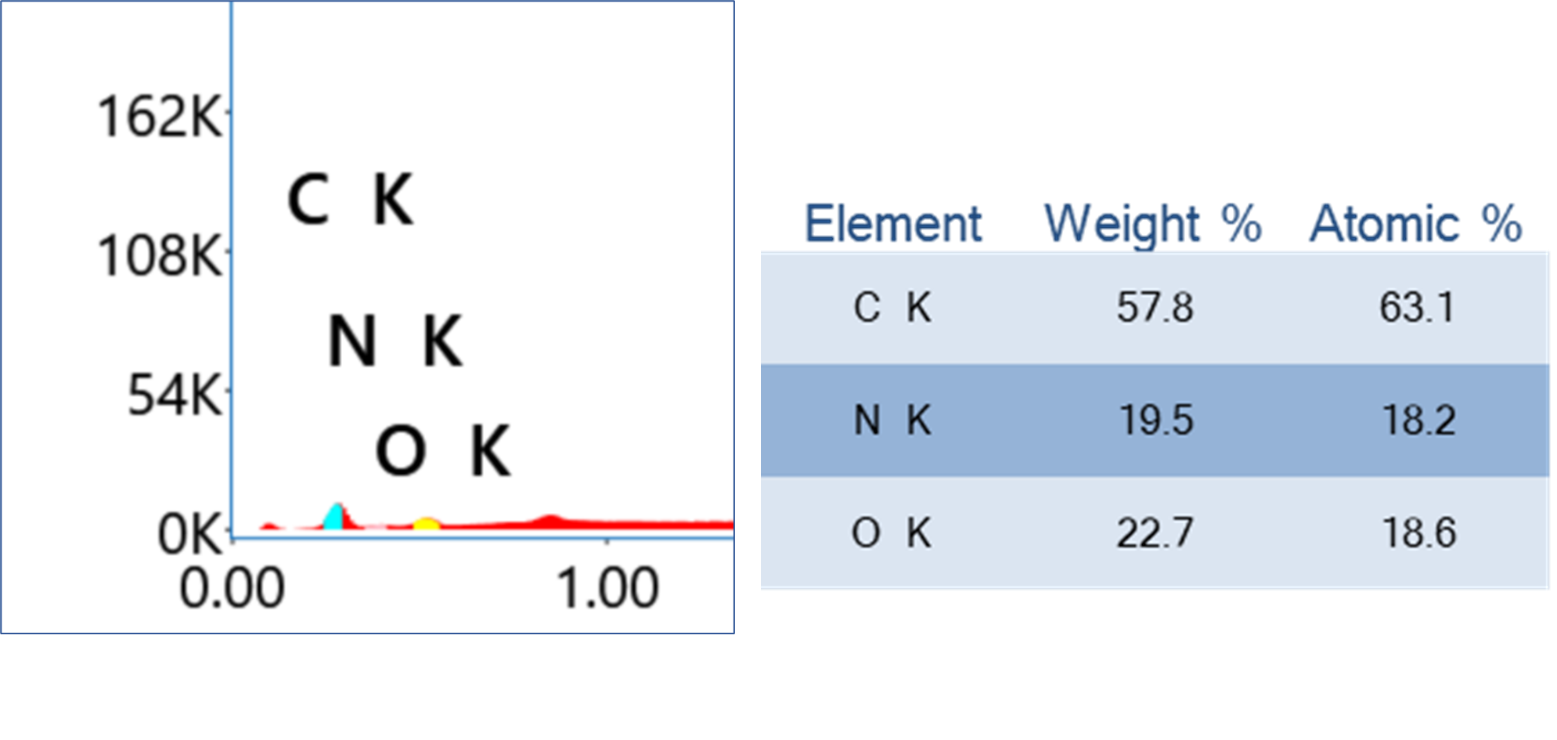
 **Figure S10.** Atomic percentage of TFPh_DHPh_COF.

**Figure S11**. (a) CV curves of as synthesized TFPh_DHPh_COF electrocatalyst at different scan rates in O_2_-saturated alkaline electrolyte solution (b) Linear fitting of capacitive currents of the TFPh_DHPh_COF electrocatalyst vs scan rate.

**Figure S12**. (a) Electrochemical impedance spectroscopy (EIS) spectra of TFPh_DHPh_COF and (b) equivalent circuit of TFPh_DHPh_COF material.

**Figure S13**. LSV curves of as synthesized TFPh_DHPh_COF electrocatalyst at different rotations in O_2_-saturated alkaline electrolyte solution.

**Figure S14**. LSV curves of as synthesized TFPh_DHPh_COF electrocatalyst with ring and disk current in O_2_-saturated alkaline electrolyte solution.

**
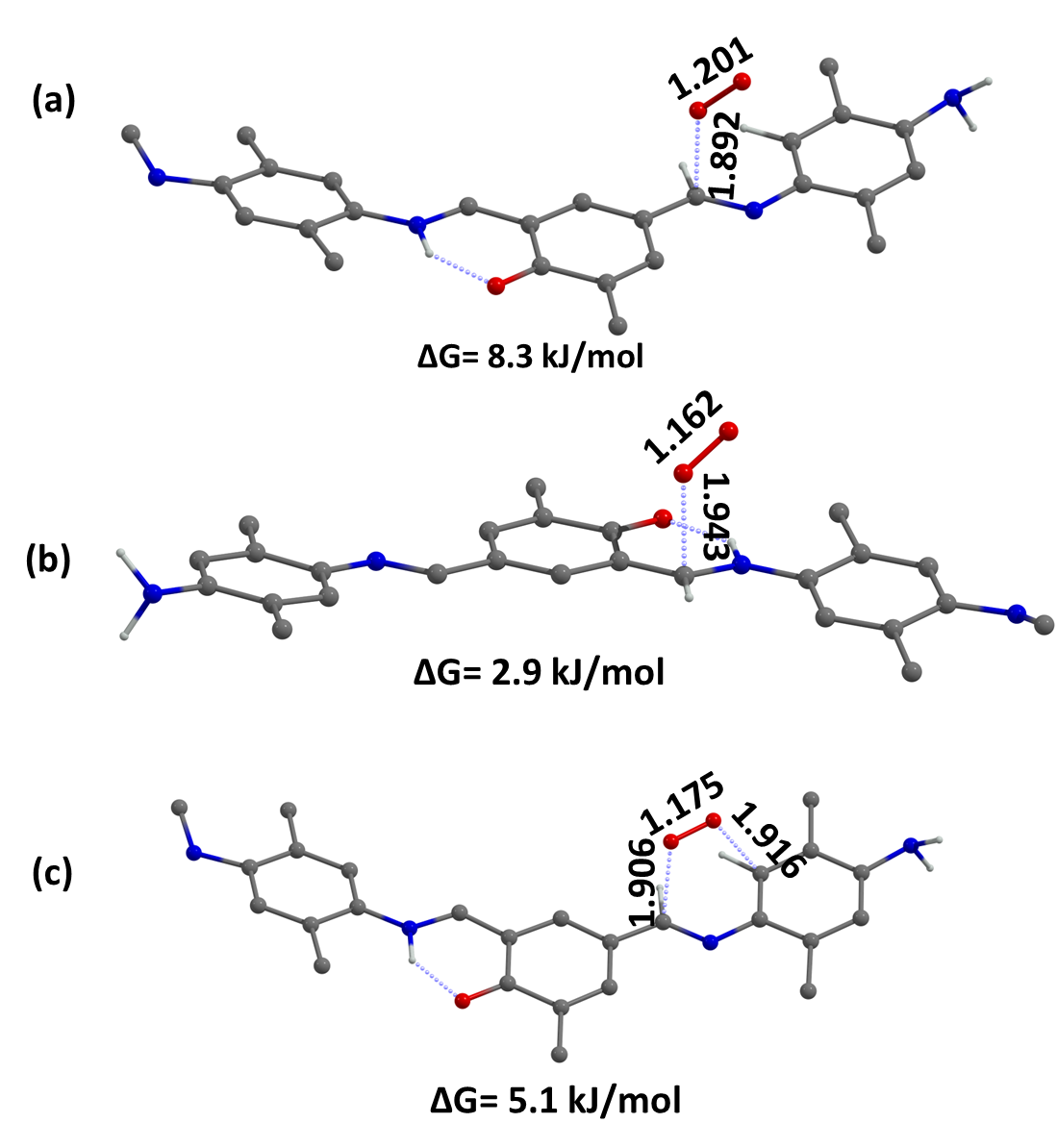
**

**Figure S15**. Comparative Gibbs free energy calculations for O₂ binding revealed that the methyl-substituted COF (TFPh_DMePh_COF).

**
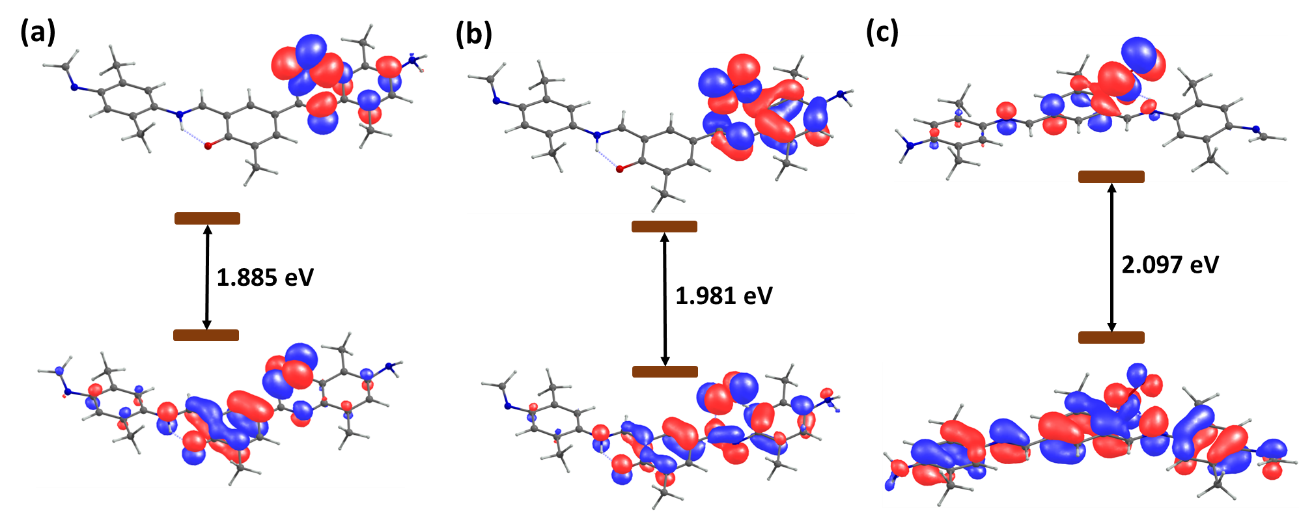
 Figure S16**. HOMO–LUMO gap of the TFPh_DHPh_COF catalysts with dual binding sites interacting with O_2_

**Table S1:** Unit cell parameters and fractional atomic coordinates for TFPh_DHPh_COF

| Space group: P1 | | | |
| --- | --- | --- | --- |
| Calculated unit cell: Triclinic | | | |
| atoms | x | y | z |
| C1 | 0.15300 | -0.85105 | -0.13635 |
| C2 | 0.23662 | -0.80038 | -0.05957 |
| C3 | 0.26551 | -0.72179 | 0.00297 |
| C4 | 0.21929 | 0.69200 | -0.05370 |
| C5 | 0.14600 | -0.73236 | -0.13595 |
| C6 | 0.10537 | -0.81746 | -0.19896 |
| N7 | 0.25028 | -0.61902 | -0.02596 |
| C8 | 0.32467 | -0.57067 | 0.03198 |
| C9 | 0.35410 | -0.50269 | 0.06103 |
| C10 | 0.43206 | -0.45805 | 0.10680 |
| C11 | 0.46639 | -0.38053 | 0.10024 |
| C12 | 0.42509 | -0.35168 | 0.07661 |
| C13 | 0.35149 | -0.39177 | 0.05412 |
| C14 | 0.31282 | -0.46915 | 0.04241 |
| C15 | 0.31807 | -0.35532 | 0.04168 |
| N16 | 0.24715 | -0.38731 | 0.02886 |
| C17 | 0.21401 | -0.34843 | 0.01429 |
| C18 | 0.25427 | -0.27424 | -0.00695 |
| C19 | 0.22222 | -0.23614 | -0.00695 |
| C20 | 0.14806 | -0.27260 | -0.01449 |
| C21 | 0.10815 | -0.34669 | 0.00717 |
| C22 | 0.14058 | -0.38456 | 0.02085 |
| N23 | 0.11137 | -0.23732 | -0.02341 |
| C24 | 0.14275 | -0.16575 | -0.06676 |
| C25 | 0.10598 | -0.13325 | -0.06815 |
| C26 | 0.14406 | -0.05452 | -0.12320 |
| C27 | 0.10591 | -0.01955 | -0.11542 |
| C28 | 0.02964 | -0.06579 | -0.05485 |
| C29 | -0.00520 | -0.14464 | -0.01080 |
| C30 | 0.03400 | -0.17433 | -0.01704 |
| C31 | -0.07684 | -0.19185 | 0.03341 |
| C32 | 0.14156 | 0.05179 | -0.16245 |
| C33 | 0.53922 | -0.33358 | 0.10708 |
| N34 | 0.58417 | -0.35574 | 0.12539 |
| O35 | 0.24830 | -0.50437 | 0.01803 |
| O36 | 0.33604 | -0.67908 | 0.02923 |
| O37 | 0.10013 | -0.45696 | 0.04179 |
| O38 | -0.00416 | -0.03959 | -0.04288 |
| O39 | 0.03899 | -0.85674 | -0.26470 |
| N39 | 0.11199 | 0.07282 | -0.10685 |
| C40 | 0.65896 | -0.30861 | 0.11774 |
| N41 | 0.87953 | -0.16946 | 0.03965 |
| C42 | 0.80545 | -0.21701 | 0.07567 |
| C43 | 0.76329 | -0.18904 | 0.06283 |
| C44 | 0.69042 | -0.23462 | 0.08208 |
| C45 | 0.70128 | -0.33666 | 0.13620 |
| C46 | 0.77401 | -0.29104 | 0.11391 |
| O47 | 0.67165 | -0.40861 | 0.17001 |
| H48 | 0.45135 | 0.70768 | 0.07497 |
| H49 | 0.27002 | 0.17349 | -0.00932 |
| H50 | 0.12364 | 0.28592 | 0.00593 |
| H51 | 0.65643 | 0.78863 | 0.06865 |
| H52 | 0.80794 | 0.68567 | 0.12699 |
| H53 | 0.31411 | 0.75578 | -0.01198 |
| H54 | 0.04833 | 0.62301 | 0.01388 |
| H55 | 0.56262 | 0.72561 | 0.09713 |
| H56 | 0.36303 | 0.41068 | 0.05639 |
| H57 | 0.35077 | 0.70447 | 0.04200 |
| H58 | 0.20168 | 0.87047 | -0.10414 |
| H59 | 1.00470 | 0.76494 | 0.02202 |
| H60 | 1.20500 | 1.07717 | 0.98179 |
| O61 | 0.79680 | 0.88524 | 1.04865 |
| O62 | 2.04725 | 1.13686 | 0.91374 |
| O63 | 1.26669 | 1.84382 | -0.07040 |
| H64 | 1.20337 | 1.98248 | -0.15431 |
| H65 | 1.46329 | 1.51919 | 0.11013 |

| **S. No.** | **Catalyst** | **Onset potential**  **E_onset_ (V)** | **Half-wave potential**  **E_1/2_ (V)** | **Limiting current density J_L_ (mA cm^-2^ )** | **Ref** |
| --- | --- | --- | --- | --- | --- |
| **1** | TFPh_DHPh_COF | **0.84** | **0.72** | **4.2** | **This work** |
| 2 | N-doped holey graphitic carbon material | Similar to Pt/C | 0.78 V |  | ^[2]^ |
| 3 | Pt-COF _800_ | 1.00 V | 0.88 | 6.79 | ^[3]^ |
| 4 | TP–BPY–COF _800_ | 0.78 V | 0.70 | 5.50 | ^[4]^ |
| 5 | TRIPTA COF | 0.77 | 0.68 | 3.1 | ^[5]^ |
| 6 | NDI0.17-COF/ SuperP | 0.769 | - | 4.2 | ^[6]^ |
| 7 | im-PY-BPY-COF | 0.92 | 0.80 | 5.8 | ^[7]^ |
| 8 | TAPP-x-COF | 0.80 | 0.66 | 4.82 | ^[8]^ |
| 9 | DAF-COF | 0.89 | 0.74 | - | ^[9]^ |
| 10 | Azo-COF | 0.88 | 0.68 | - | ^[10]^ |
| 11 | JUC-608 | 0.84 | 0.72 |  | ^[11]^ |
| 12 | CTFs | 0.75 | 0.60 |  | ^[12]^ |
| 13 | COF-JLU-82  COF-JLU-23 | 0.98 V  0.99 V | 0.68 V  0.66 V |  | ^[13]^ |
| 14 | PA@TAPT-DHTACOF1000NH3 | 0.98 | 0.87 | 7.2 | ^[14]^ |
| 15 | RCOF | - | 0.82 | 5.63 | ^[15]^ |

**Table S2:** Unit cell parameters and fractional atomic coordinates for TFPh_DHPh_COF

**References**

[1] A. Kastrati, C. G. Bochet, *Journal of Organic Chemistry* **2019**, *84*, 7776–7785.

[2] C. Y. Lin, D. Zhang, Z. Zhao, Z. Xia, *Advanced Materials* **2018**, *30*, 1703646.

[3] X. Li, S. Yang, M. Liu, S. Liu, Q. Miao, Z. Duan, P. Qiao, J. Lin, F. Sun, Q. Xu, Z. Jiang, *Small Struct* **2023**, *4*, 2200320.

[4] X. Li, S. Yang, M. Liu, S. Liu, Q. Miao, Z. Duan, P. Qiao, J. Lin, F. Sun, Q. Xu, Z. Jiang, *Small Struct* **2023**, *4*, 2200320.

[5] S. K. Das, G. Kumar, M. Das, R. S. Dey, *Mater Today Proc* **2022**, *57*, 228–233.

[6] M. Martínez-Fernández, E. Martínez-Periñán, J. I. Martínez, M. Gordo-Lozano, F. Zamora, J. L. Segura, E. Lorenzo, *ACS Sustain Chem Eng* **2023**, *11*, 1763–1773.

[7] X. Yang, Q. An, X. Li, Y. Fu, S. Yang, M. Liu, Q. Xu, G. Zeng, *Nature Communications 2024 15:1* **2024**, *15*, 1–10.

[8] X. Liang, Z. Zhao, R. Shi, L. Yang, B. Zhao, H. Qiao, L. Zhai, *Molecules* **2023**, *28*, 4680.

[9] Z. You, B. Wang, Z. Zhao, Q. Zhang, W. Song, C. Zhang, X. Long, Y. Xia, *Advanced Materials* **2023**, *35*, 2209129.

[10] X. Yan, B. Wang, J. Ren, X. Long, D. Yang, *Angewandte Chemie* **2022**, *134*, e202209583.

[11] S. Chang, C. Li, H. Li, L. Zhu, Q. Fang, *Chem Res Chin Univ* **2022**, *38*, 396–401.

[12] J. Liu, Y. Hu, J. Cao, *Catal Commun* **2015**, *66*, 91–94.

[13] J. Jia, J. Li, S. Ma, Z. Zhang, X. Liu, *Macromol Rapid Commun* **2023**, *44*, 2200717.

[14] Q. Xu, Y. Tang, X. Zhang, Y. Oshima, Q. Chen, D. Jiang, *Advanced Materials* **2018**, *30*, 1706330.

[15] T. Xu, H. Zhou, X. Zhang, T. S. Herng, J. Ding, C. Chi, J. Zhu, *Angewandte Chemie* **2025**, *137*, e202424449.
